# NaHS immersion alleviates the stress effect of chromium(III) on alfalfa seeds by affecting active oxygen metabolism

**DOI:** 10.1080/15592324.2024.2375673

**Published:** 2024-07-07

**Authors:** Ting Bu, Jianxia Yang, Jianxin Liu, Xiaofeng Fan

**Affiliations:** a University Provincial Key Laboratory for Protection and Utilization of Longdong Bio-Resources in Gansu Province, Qingyang, Gansu Province, China; b School of Agriculture and Bioengineering, Longdong University, Qingyang, China

**Keywords:** H_2_S, Cr(iii), alfalfa, ROS, GSH

## Abstract

**Objective:**

This study aimed to investigate the regulatory effects of exogenous hydrogen sulfide (H_2_S) on seed germination, seedling growth, and reactive oxygen species (ROS) homeostasis in alfalfa under chromium (Cr) ion (III) stress.

**Methods:**

The effects of 0–4 mM Cr(III) on the germination and seedling growth of alfalfa were first assessed. Subsequently, following seed NaHS immersion, the influence of H_2_S on alfalfa seed germination and seedling growth under 2 mM Cr(III) stress was investigated, and the substance contents and enzyme activities associated with ROS metabolism were quantified.

**Results:**

Compared to the control group, alfalfa plant germination was delayed under 2 mM Cr(III) stress for up to 48 h (*p* < 0.05). At 120 h, the total seedling length was approximately halved, and the root length was roughly one-third of the control. Treatment with 0.02–0.1 mM NaHS alleviated the delay in germination and root growth inhibition caused by 2 mM Cr(III) stress, resulting in an increased ratio of root length to hypocotyl length from 0.57 to 1 above. Additionally, immersion in 0.05 mM NaHS reduced hydrogen peroxide (H_2_O_2_) and oxygen-free radicals (O_2_^· –^) levels (*p* < 0.05), boosted glutathione (GSH) levels (*p* < 0.05), and notably enhanced catalase (CAT), ascorbate peroxidase (APX), and glutathione reductase (GR) activities (*p* < 0.05) compared to the 2 mM Cr(III) stress treatment group.

**Conclusion:**

Seed immersion in NaHS mitigated the delay in germination and inhibition of root elongation under 2 mM Cr(III) stress. This effect is likely attributed to the regulation of intracellular ROS homeostasis and redox balance through enzymatic and non-enzymatic systems; thus, providing a potential mechanism for combating oxidative stress.

## 1. Introduction

Alfalfa (*Medicago sativa* L.) is widely grown as a forage species due to its excellent tolerance to saline‒alkali conditions and adaptability, making it a preferred choice for cultivation.^1^ It has been extensively utilized and shows significant potential for soil remediation and environmental protection.^2,3^ However, in Cr(III)-contaminated soil，with a pH exceeding four, Cr(III) tends to become less soluble form (CrOH_2t_, Cr(OH)_2_*, Cr(OH)_3_) and bind with organic materials as the pH increases.^4^ Further, in the presence of reducing agent, Cr(III) in turn forms stable soluble complexes in acidic conditions.^4^ This occurrence adversely affects the normal growth and development of plants like alfalfa, compromising their overall vigor and productivity.^5–7^ Compared to plants exposed to Cr(VI), rice seedling tissues exhibit a higher capacity for Cr accumulation under exposure Cr(III).^8^ These factors should be considered when cultivating alfalfa in potentially Cr-contaminated areas. Further research into methods to mitigate Cr(III) impact on alfalfa growth could ensure its continued success as a valuable forage crop.

Currently, scientists are exploring innovative approaches to mitigate nonbiological stress effects on plants.^9–11^ Seed priming can improve seed germination and seedling establishment in response to harsh environmental conditions, including significant abiotic and biotic stresses.^12^ H_2_S signaling molecules have been shown to induce stress tolerance by modulating antioxidant activity, reducing glutathione levels, increasing osmotic regulator accumulation, and enhancing expression of stress-related genes and cell signaling proteins.^13,14^ Pretreating Arabidopsis with H_2_S before drought stress regulates anthocyanin, proline, and hydrogen peroxide levels, amino acid metabolism, autophagy, protein ubiquitination, and redox homeostasis via protein persulfidation.^15^ Under salt stress, the H_2_S-regulated protein persulfidation enhances the activity of Glucose-6-phosphate dehydrogenases (G6PDs) in both Arabidopsis and tomato cell sap by inducing structural changes in the homotetrameric form of cytosolic G6PD, thereby mitigating oxidative damage.^16^ Under toxic metal stress, H_2_S modifies metal transporter proteins, enhances antioxidant capacity, and participates in protein S-sulfhydration and microRNA activity to counteract toxic metal stress.^17^ NaHS, an exogenous H_2_S donor, partially alleviates Cr(VI) stress-induced growth inhibition in *Zea mays* L., indicating the protective role of H_2_S signaling in plants facing heavy metal contamination.^18^ Additionally, H_2_S reduces lipid peroxidation, improves growth inhibition, and alleviates metal toxicity in alfalfa plants exposed to lead (Pb) and cadmium (Cd) stress,^19,20^ highlighting its potential to enhance plant health and resilience to environmental stressors. These findings underscore the potential of H_2_S as a natural remedy to mitigate heavy metal contamination’s adverse effects on plant growth and development.

It was hypothesized that H_2_S could potentially mitigate adverse effects and improve the germination and growth of alfalfa seeds by regulating ROS metabolism. To test this hypothesis and understand the underlying mechanism, alfalfa seeds were exposed to various concentrations of Cr(III) solution, and their germination and seedling growth were observed. Subsequently, different doses of the H_2_S donor NaHS were applied to evaluate the germination rate and root elongation of the seeds, while also assessing ROS production and the response of both antioxidant enzyme systems and non-enzymatic systems. These findings are expected to offer scientific evidence supporting the use of H_2_S to enhance alfalfa growth in Cr(III)-contaminated soils.

## 2. Materials and methods

### 2.1 Materials

The variety of alfalfa utilized in this study was *Medicago sativa* L. cv. Victoria, which was procured from the Seed Station of Xifeng District, Qingyang City, China. The source of Cr(III) was CrCl_3_**·**6 H_2_O (Kermel, Tianjin, China), and the H_2_S signaling molecule donor was NaHS (Urchem, Shanghai, China).

## 2.2 Methods

### 2.2.1 Seed pretreatment and planting

Alfalfa seeds that were fully grained and free of disease and pests were selected, cleaned, and sterilized with 2% NaClO for 10 min, immersed in purified water for 12 h, and subjected to two-dimensional paper chromatography with different concentrations of Cr(III) (0, 0.5, 1, 2, 3, and 4 mM) in germination boxes (12 cm × 12 cm ×6 cm), to measure the stress effect of Cr(III). A Cr(III) concentration of 0 was used as a control.

After cleaning and disinfection, the alfalfa seeds were immersed in different concentrations of NaHS (0, 0.02, 0.05, 0.08, 0.10, 0.15, or 0.20 mM) for 12 h and subsequently subjected to two-dimensional paper chromatography in germination boxes containing 2 mM Cr(III) to determine the effect of NaHS immersion on Cr(III) stress. In control samples, NaHS and 2 mM Cr(III) were replaced with distilled water without any treatment.

In every experiment, a total of 50 seeds were sown in each box, with four replicate samples for each treatment. Following planting, the plants were placed in an incubator (BOXUN, GSP-9080MBE, China) at a temperature of 25 ± 0.5°C, humidity of 60% and no light for 48 h, after which they were transferred to an illuminated incubator (GXM, GXM-358C-3, China) with a light intensity of 200 µmol m^−2^ s^−1^, a photoperiod of 14 h, and appropriate daily watering. The humidity was maintained at 60%. The relevant indices were measured at the end of the fifth day, and the experiments were repeated 3 times.

### 2.2.2 Determination of alfalfa seed germination

Using a radicle breaking 1 mm through the seed coat as the germination standard, the growth of the seeds was monitored at 12-hour intervals, and the number of sprouted seeds was recorded at each time point to calculate the germination rate (germination rate = number of germinated seeds/total number of seeds) over a period of 84 h. After 120 h (5 days), photographs were taken, and the total seedling length and root length were measured. Additionally, the ratio of root length to hypocotyl length (ratio = root length/total seedling length) was calculated. All photographs were captured using a Power Shot SX30IS digital camera.

### 2.2.3 Determination of ROS and peroxidation products of membrane lipids

The concentration of H_2_O_2_ was determined using the xylenol orange method described by Gay et al.^21^ O_2_^· –^ was measured following the method developed by Elstner et al.^22^ Malondialdehyde (MDA) was quantified employing the thiobarbituric acid (TBA) assay outlined by Yang et al.^23^

### 2.2.4 Determination of enzyme activity and substance content of the ROS scavenging system

The activities of superoxide dismutase (SOD), CAT, peroxidase (POD), APX, and GR were determined following the methods outlined in the studies by Amooaghaie,^24^ Foster^25^ and Oloumi.^10^ Alfalfa seedlings (0.5 g) were weighed, homogenized with 5 mL of phosphate buffer (50 mM PBS, pH 7.8) on ice, and then centrifuged at 10,000 × g for 15 min at 4°C. The resulting supernatant was collected to evaluate the activities of SOD, CAT, POD, and GR. SOD activity was assessed using the NBT photoreduction method at 560 nm, with one unit of enzyme activity defined as 50% inhibition of NBT photoreduction. CAT activity was measured at 240 nm by monitoring H_2_O_2_ decomposition, where a decrease in OD of 0.01 per minute was defined as one unit of enzyme activity. POD activity was determined by measuring the increase in OD at 470 nm due to guaiacol oxidation in the presence of H_2_O_2_, with one unit of enzyme activity corresponding to an OD increase of 0.01 per minute. GR enzyme activity was evaluated at 340 nm, with one unit of GR defined as the rate of NADPH oxidation and absorbance decrease at 1 µmol min^−1^. These enzyme activities are expressed in units of U·g^−1^ FW.

Alfalfa seedlings (0.5 g) were weighed and homogenized by adding 5 mL of phosphate buffer (50 mM K_2_HPO_4_-KH_2_PO_4_, pH 7.0, containing 2 mmol/L ascorbic acid (AsA) and 0.1 mmol/L EDTA-Na_2_) on ice and then centrifuged at 10,000×g for 15 min at 4°C. Of supernatant, 100 µL was mixed with 1.9 mL of solution (50 mM PBS, pH 7.0, 0.3 mmol/L AsA), and then 1 mL of H_2_O_2_ was added immediately. The change in OD at 290 nm was measured to calculate the APX enzyme activity.

The GSH content was measured using the dinitrobenzoic acid method,^26^ and the determination of AsA was based on the method proposed by Jin et al.^27^ Soluble sugars were quantified using the anthrone colorimetric method;^28^ soluble protein content was assessed by the Coomassie brilliant blue method;^29^ and proline content was evaluated through a tninhydrin chromogenic assay.^30^

### 2.3 Data processing

SPSS version 20.0 was used for one-way ANOVA. For post hoc testing, Duncan’s multiple range test (DMRT) was utilized with a significance level of *p* < 0.05 to determine statistically significant differences among groups. Each treatment had 4 independent biological replicates (*n* = 4).

## 3. Results

### 3.1 Effects of Cr(III) on alfalfa seed germination and seedling growth

The statistical outcomes for alfalfa seed germination under Cr(III) stress are detailed in Table 1. At a concentration of 0.5 mM, the germination rate surpassed that of the control group from 24 to 84 h, indicating a promotion of germination by Cr. However, at concentrations of 1 mM and 2 mM, the germination rate was lower than the control’s before 48 h, indicating a delay in effect. Additionally, at 3 mM concentration, the seed germination rate remained lower than the control’s until 72 h, indicating an intensified delay effect. Exposure to 4 mM Cr resulted in delayed seed germination, with a final germination rate significantly lower than that of the control group.

**Table 1.** Germination of alfalfa seeds under 0–4 mM Cr(III) stress for 84 h (germination percentage, %).

Cr(III) concentration (mM)	Time (hours)						
	12	24	36	48	60	72	84
0	2.7 ± 1.1a	55.2 ± 5.7a	74.4 ± 8.0a	76.6 ± 6.9a	77.3 ± 2.9a	81.4 ± 9.3a	86.9 ± 4.9a
0.5	1.4 ± 1.2a	56.0 ± 8.0a	78.1 ± 8.4b	81.6 ± 4.6b	83.8 ± 5.0b	84.5 ± 4.1b	86.6 ± 4.3a
1	3.8 ± 1.5a	43.1 ± 4.8b	67.5 ± 3.0c	72.1 ± 1.1ac	80.1 ± 5.2ab	85.8 ± 6.9b	85.8 ± 6.9a
2	2.9 ± 1.2a	40.0 ± 1.2bc	67.3 ± 7.6c	76.2 ± 4.2a	81.5 ± 1.1b	84.2 ± 4.4b	84.5 ± 3.5a
3	1.5 ± 1.3a	31.0 ± 3.4d	53.0 ± 10.9d	68.3 ± 9.5d	69.4 ± 9.3c	74.3 ±14.6c	86.8 ±0.7a
4	2.4 ± 0.1a	9.3 ± 6.2e	37.4 ± 11.4e	51.6 ± 7.4e	56.1 ± 13.8d	64.8 ± 4.9d	64.8 ± 4.9b

Note: Data are presented as the mean ±SD (*n* = 3), and distinct lowercase letters within the same column indicate statistically significant differences (*p* < 0.05). The same as below.

**Figure 1.**
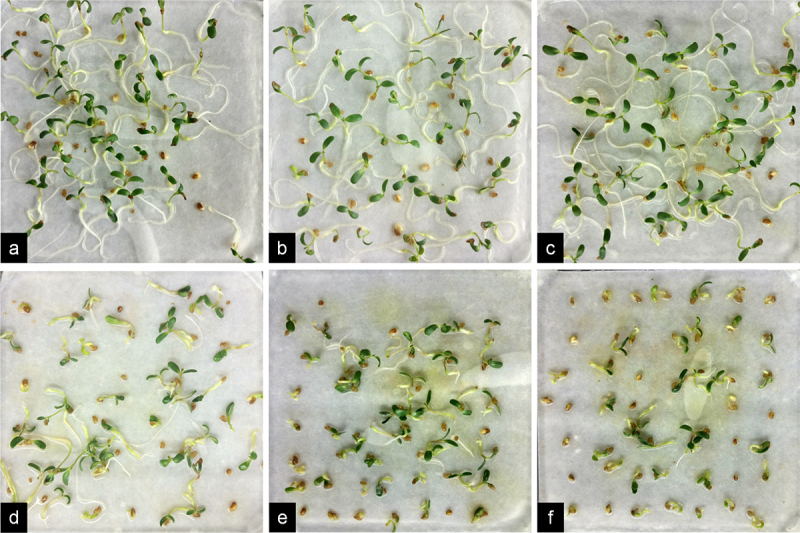
Seedling growth of alfalfa plants under 0–4 mM Cr(III) stress for 5 days after planting. a. 0 mM; b. 0.5 mM; c. 1 mM; d. 2 mM; e. 3 mM; f. 4 mM.

Regarding seedling growth, there were no notable differences in the growth of alfalfa seedlings compared to the control group when Cr concentrations were 0.5 mM and 1 mM. The plants exhibited good germ and radicle growth along with two cotyledons (Figure 1a,b). However, at Cr concentrations ≥2 mM, there was a significant decrease in radicle length, and the radicle turned green. With increasing Cr concentration, there was a rise in the number of ungerminated seeds (Figure 1). At a Cr concentration of 4 mM, most seeds germinated, but only a few developed radicles (Figure 1f).

**Figure 2.**
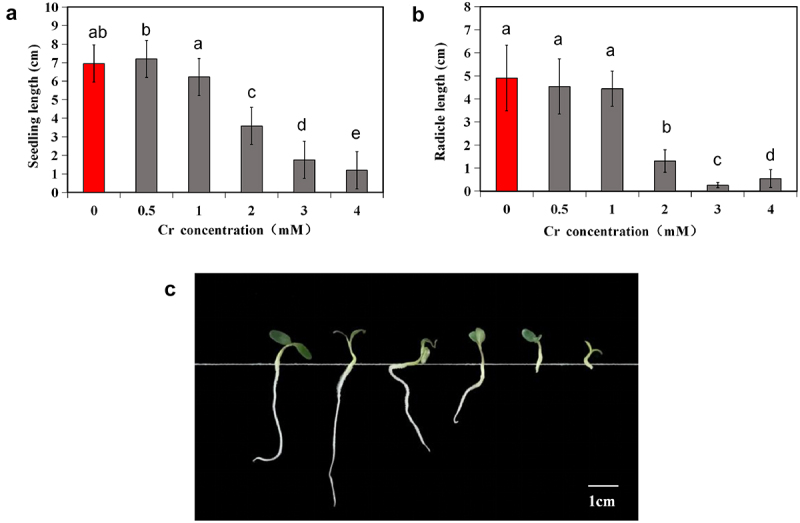
Seedling length (a), (b) and comparison of root and Hhypocotyl morphology (c) of alfalfa under 0–4 mM Cr(III) stress for 5 days after seed planting. The different lower case letters above the columns indicate significant differences among the treatments (*p* <0.05), and the Cr concentrations from left to right are 0, 0.5, 1, 2, 3, and 4 mM in Panel C.

Total length and radicle length were quantified (Figure 2). At a Cr stress concentration of 0.5 mM, seedlings showed greater total length than those in the control group, while radicle length decreased. This suggests that 0.5 mM Cr primarily promoted seedling growth through hypocotyl elongation, while root elongation was hindered. At concentrations ≥2 mM, plant growth notably declined, with significant decreases in total length and root elongation (*p* < 0.05). The total length and root length were approximately half and one-third of those in the control group, respectively, at a 2 mM Cr concentration (Figure 2a,b). Further examination revealed that at concentrations of 3 mM and 4 mM, roots were very short, and root tips were blunt and round (Figure 2c).

### 3.2 Effects of exogenous H_2_S on the germination and seedling growth of alfalfa under 2 mM Cr(III) stress

Seeds were pretreated with the H_2_S donor NaHS before exposure to 2 mM Cr(III). Analysis of seed germination dynamics suggested that, compared with that in the control group, the seed germination rate in the 0.02–0.15 mM NaHS pretreatment group was consistently greater than that in the 2 mM Cr(III) stress group at each time point, suggesting that exogenous H_2_S could mitigate the inhibitory effect of Cr(III) stress on seed germination. Notably, the 0.05 mM NaHS treatment had the most pronounced effect (Table 2).

**Table 2.** Effect of NaHS on the germination of alfalfa plants under 2 mM Cr(III) stress within 84 h (germination percentage, %).

NaHS concentration (mmol·L-1)	2 mM Cr (III)	Time (hour)						
		12	24	36	48	60	72	84
0	–	2.7 ± 1.2a	56.0 ± 3.5a	74.7 ± 3.1a	77.3 ± 4.2a	79.3 ± 4.2a	82.0 ± 3.5a	87.3 ± 1.2a
0	+	2.7 ± 1.2a	38.0 ± 11.4b	67.3 ± 4.0b	76.0 ± 3.5a	80.7 ± 3.1a	84.7 ± 1.2ab	84.7 ± 1.2b
0.02	+	2.8 ± 0.0a	38.7 ± 13.3b	73.0 ± 9.0a	81.3 ± 5.0b	84.8 ± 2.3b	86.2 ± 1.2b	86.9 ± 2.0ab
0.05	+	2.0 ± 1.2a	42.0 ± 2.0c	73.3 ± 6.4a	81.3 ± 4.0b	86.7 ± 1.2b	88.7 ± 1.2b	89.3 ± 1.2a
0.08	+	2.7 ± 1.2a	45.3 ± 2.0d	70.7 ± 8.3c	80.0 ± 5.3b	85.3 ± 3.1b	85.3 ± 2.3b	86.0 ± 2.3ab
0.10	+	1.3 ± 1.2a	40.0 ± 3.1bc	72.0 ± 6.1ac	81.3 ± 3.1b	82.7 ± 3.1bc	85.3 ± 4.0b	85.3 ± 3.1ab
0.15	+	2.7 ± 1.1a	43.3 ± 5.1c	68.7 ± 8.0b	82.0 ± 5.1b	83.3 ± 4.3bc	86.7 ± 5.6b	86.7 ± 4.5ab
0.2	+	1.3 ± 2.0a	37.3 ± 6.5b	63.3 ± 8.0d	77.3 ± 5.1a	82.0 ± 5.4ac	82.7 ± 6.6a	84.0 ± 5.4b

**Figure 3.**
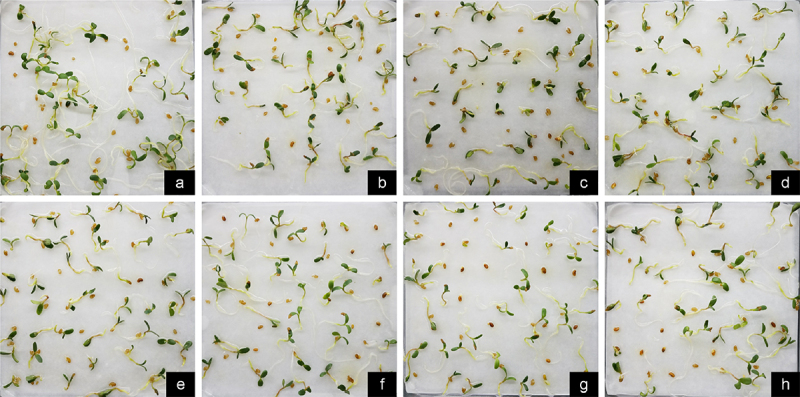
Effect of exogenous H_2_S on the growth of alfalfa plants treated with 2 mM Cr(III) for 5 days after planting. a. CK; b. 2 mM Cr; c. 2 mM Cr +0.02 mM NaHS; d. 2 mM Cr +0.05 mM NaHS; e. 2 mM Cr +0.08 mM NaHS; f. 2 mM Cr +0.10 mM NaHS; g. 2 mM Cr +0.15 mM NaHS; h. 2 mM Cr +0.2 mM NaHS.

**Figure 4.**
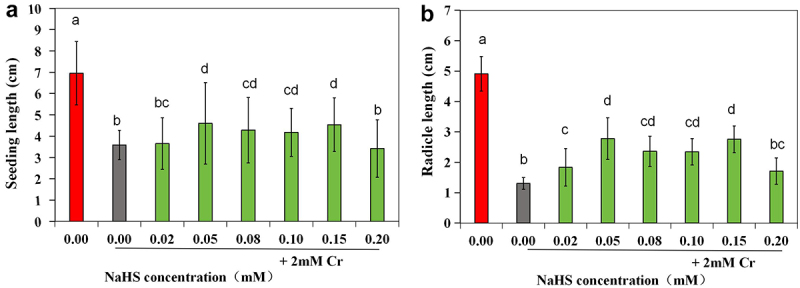
Effects of exogenous H_2_S on seedling length (a) and radicle length (b) of alfalfa plants treated with 2 mM Cr(III) for 5 days after seed planting. The x-axis represents each treatment group. The different lowercase letters above the columns indicate significant differences among the treatments (*p* < 0.05), as indicated below.

Upon thorough examination of the results depicted in Figure 3, it was noted that treating seeds with NaHS at concentrations ranging from 0.02 to 0.10 mM before exposure to Cr stress resulted in enhanced seedling growth compared to stress alone. Subsequent analysis of the data on seedling length and radicle root length indicated that the total length of plants treated with NaHS at concentrations between 0.05 and 0.15 mM prior to Cr stress treatment was notably greater than that of plants subjected solely to stress (*p* < 0.05) (Figure 4a). Additionally, radicle length was significantly greater with pretreatment of ≥0.02 mM NaHS compared to single stress conditions (*p* < 0.05) (Figure 4b). The ratio of radicle root length to hypocotyl length was only 0.57 under the 2 mM Cr treatment (Table 3), lower than that of the control, indicating inhibition of root elongation by Cr(III); however, this ratio increased under exogenous H_2_S treatment, with the effects of the 0.05 and 0.15 mM NaHS treatments being most pronounced. These findings suggest that exogenous H_2_S has the potential to alleviate the inhibitory effects of Cr stress on root elongation.

**Table 3.** Effect of NaHS immersion on the root length:hypocotyl ratio of alfalfa seedlings under 2 mM Cr(III) stress.

NaHS concentration (mM)	CK	0	0.02	0.05	0.08	0.1	0.15	0.2
Root length: hypocotyl (ratio)	2.41	0.57	1.01	1.53	1.23	1.28	1.55	1.00

### 3.3 Effects of exogenous H_2_S on O_2_^· –^, H_2_O_2_ and MDA contents under 2 mM Cr(III) stress

**Figure 5.**
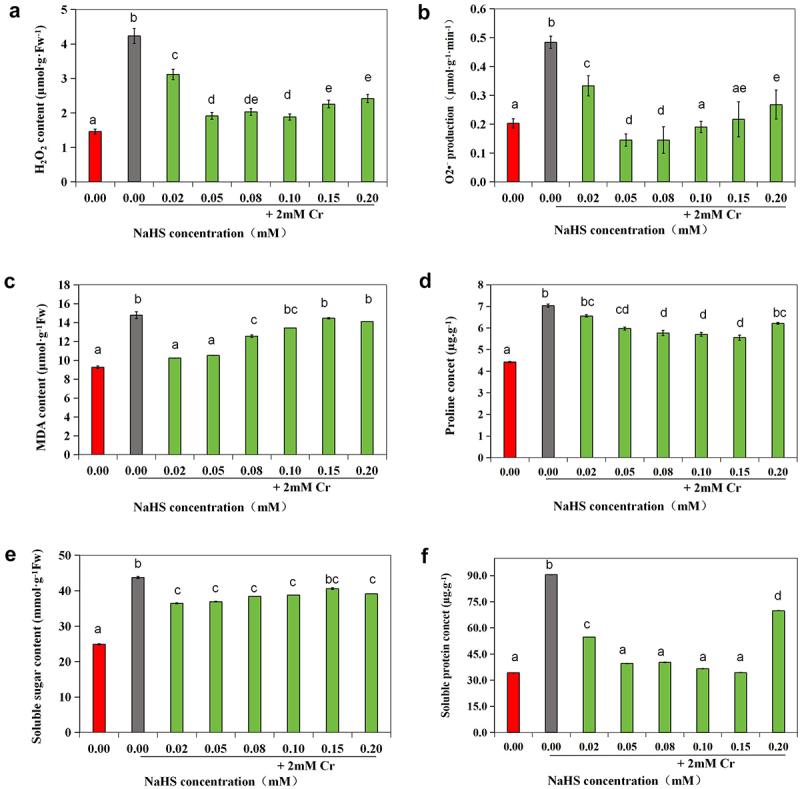
Effects of exogenous H_2_S on O_2_^· –^(a), H_2_O_2_ (b), MDA (c), proline (d), soluble sugar (e) and soluble protein (f) content in alfalfa under 2 mM Cr(III) stress for 5 days after seed planting.

Compared to the control group, there was a noticeable increase in H_2_O_2_ and O_2_^· –^ levels under 2 mM Cr(III) stress (*p* < 0.05). Additionally, MDA, proline, soluble sugar, and soluble protein levels were significantly elevated (*p* < 0.05). Furthermore, compared to the Cr(III) stress group, H_2_O_2_ and O_2_^· –^ levels significantly decreased in seeds treated with 0.05 mM and 0.08 mM NaHS (*p* < 0.05), with the most significant effect observed in these treatment groups. The MDA content notably decreased (*p* < 0.05), with no discernible difference between the 0.02 and 0.05 mM NaHS immersion groups and the control group (untreated). Proline and soluble sugar levels were higher than those in the control group (*p* < 0.05) but significantly lower than those in the Cr(III) stress group (*p* < 0.05). The soluble protein content was lower than that of the Cr(III) stress group (*p* < 0.05), with no difference observed between the Cr(III) stress group and the control group (*p* < 0.05) (Figure 5).

### 3.4 Effects of exogenous H_2_S on the enzyme activities and contents of ROS scavengers in alfalfa under 2 mM Cr(III) stress

**Figure 6.**
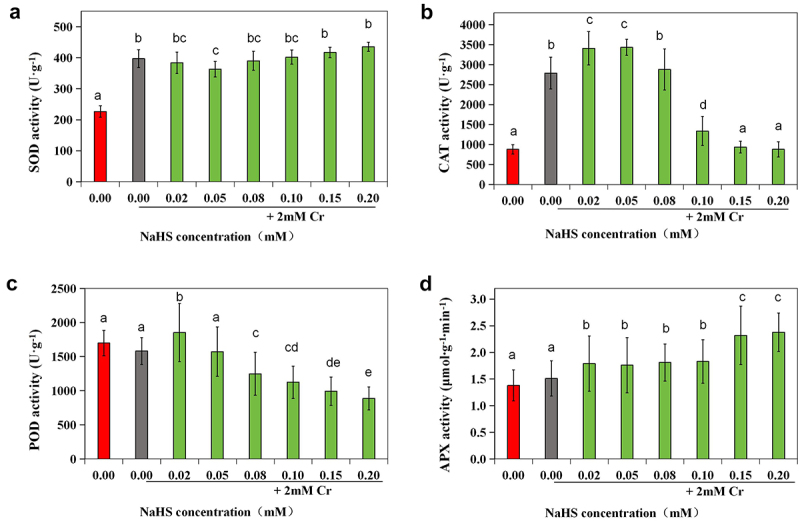
Effects of exogenous H_2_S on SOD (a), CAT (b), POD (c), and APX (d) activity in alfalfa under 2 mM Cr(III) stress for 5 days after seed planting.

**Figure 7.**
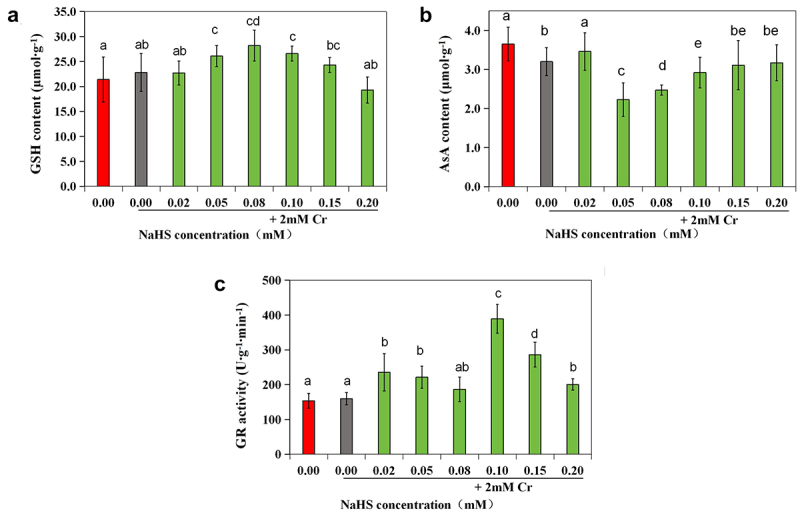
Effects of exogenous H_2_S on the GSH content (a), AsA content (b), and GR activity (c) in alfalfa under 2 mM Cr(III) stress for 5 days after seed planting.

Compared with those in the control group, the activities of SOD and CAT were significantly elevated in the 2 mM Cr(III) stress group (*p* < 0.05), while there were no notable changes in the GSH content or POD, APX, or GR enzyme activity (Figure 6). SOD activity in the 0.05 mM NaHS treatment group was significantly greater than that in the control group (*p* < 0.05) but notably lower than that in the stress group (*p* < 0.05) (Figure 6a). The activity of CAT was significantly greater in the 0.02 mM and 0.05 mM NaHS treatment groups than in the control group (*p* < 0.05) (Figure 6b). However, when the concentration of NaHS exceeded 0.05 mM, POD activity decreased with increasing NaHS concentration (Figure 6c). APX enzyme activity was also significantly different from that of the control (*p* < 0.05) (Figure 6d). Furthermore, when the seed concentration of NaHS ranged from 0.02 mM to 0.20 mM, there was an initial increase followed by a decrease in the GSH content, while the content of AsA initially decreased followed by an increase (Figure 7a,b). Additionally, compared with that in the control group, the GR enzyme activity in the 0.02 mM, 0.05 mM, 0.10 mM and 0.15 mM NaHS treatment groups significantly increased (*p* < 0.05) (Figure 7c).

## 4. Discussion

At low concentrations (≤0.5 mM), Cr(III) has been observed to enhance the growth of alfalfa seedlings, consistent with findings in wheat by Chen et al.,^31^ while not significantly affecting the growth of sorghum plants.^32^ Under 2 mM Cr(III) stress, alfalfa seed germination was delayed, resulting in a lower germination level compared to the control until 48 h. After 5 days of planting, the total seedling length was approximately half that of the control, with the root length being around one-third. Additionally, root tips showed a blunt and round shape, indicating inhibition of seed germination and impact on both root elongation and root tip development. Scoccianti et al. also noted a similar trend in celery, where the inhibitory effect of 0.01 to 1 mM Cr(III) on seed germination and hypocotyl elongation increased with concentration, with complete blockage observed at 10 mM.^33^ However, even at the lowest Cr(III) dose, celery seedling roots suffered severe damage, highlighting alfalfa’s relatively high tolerance to Cr(III).

Strategies such as neodymium and silicon have been proposed to mitigate Cr(III) stress effects and enhance plant tolerance.^7,34^ Additionally, H_2_S, a signaling molecule, has been found to boost chlorophyll and biomass levels in alfalfa^35^ and enhance growth and yield in soilless-cultivated strawberry plants.^36^ Moreover, H_2_S has been demonstrated to stimulate alfalfa hypocotyl elongation by influencing cellulose content and fibril arrangement.^37^ Treating alfalfa seeds with the exogenous H_2_S donor NaHS at concentrations of 0.02, 0.05, and 0.10 mM before planting alleviated the delay in seed germination induced by 2 mM Cr(III) stress. Even at 36 h, there was no significant difference in seed germination rate compared to the control, indicating an overall improvement. Statistical analysis revealed that exogenous H_2_S signaling boosted total and root length under Cr(III) stress, along with the ratio of root length to hypocotyl, suggesting alleviation of the inhibitory effect of 2 mM Cr(III) stress on root elongation. At NaHS concentrations of 0.05 mM and 0.15 mM, seedling total length, radicle length, and the root length to hypocotyl ratio peaked, indicating significant weakening of NaHS’s inhibitory effect on root elongation. Similarly, other studies have reported that exogenous H_2_S can alleviate growth inhibition caused by abiotic stress in plants like *Miscanthus sacchariflorus*, strawberry plants, and *foxtail millet*.^38–40^ When plants encounter metal stress, an excess of metal ions can induce osmotic stress and ion toxicity, resulting in the accumulation of ROS, which compromises cell membrane integrity.^41,42^ This triggers plants to counteract oxidative damage and protect themselves through both enzymatic pathways (such as SOD, CAT, POD, and APX) and non-enzymatic pathways (such as AsA and GSH).^6,7,43,44^ Different growth stages and metal ion stresses prompt plants to employ various defense mechanisms against ROS and maintain ROS balance.^32,45^ This study revealed that treating seeds with suitable NaHS concentrations before exposure to 2 mM Cr(III) stress reduced the accumulation of H_2_O_2_ and O_2_^· –^ induced by stress. The most significant effect was observed with NaHS concentrations between 0.05 and 0.10 mM. Under 2 mM Cr(III) stress, MDA levels increased, indicating oxidative damage to membrane lipids. Consequently, intracellular osmotic regulatory substances like proline, soluble sugars, and soluble proteins increased significantly to preserve homeostasis and shield cells from oxidative damage and other harmful factors affecting their structure and function. After treatment with 0.02 and 0.05 mM NaHS, a marked decrease in MDA levels was noted, suggesting a reduction in oxidative damage to membrane lipids. This could be attributed to increased H_2_S content following NaHS immersion, directly involved in ROS scavenging or enhancing ROS scavenging capability. Additionally, there was no significant decrease in proline or soluble sugar content, indicating tissue possessed osmoregulatory mechanisms to resist oxidative damage and protect itself.

Exposure to Cr(III) adversely affects plant growth and morphology. Excessive Cr(III) can disrupt various plant metabolic processes, leading to chlorosis, necrosis, impaired photosynthesis, and eventual mortality.^6,33^ Lin et al. demonstrated that rice plants, in the presence of sodium hydrosulfide,^45^ mitigated the impact of Cr(III) stress through both enzymatic and non-enzymatic pathways. Under Cr(III) stress, the activities of SOD, CAT, POD, and APX in alfalfa seedlings changed following NaHS immersion, with changes correlated with NaHS concentration. For instance, at a NaHS concentration of 0.02 mM, CAT, POD, and APX enzyme activities were significantly higher than in the 2 mM Cr(III) stress group (*p* < 0.05). At 0.05 mM NaHS, CAT and APX enzyme activities were also notably higher than in the 2 mM Cr(III) stress group (*p* < 0.05). These findings suggested NaHS immersion increased ROS scavenging enzyme activities under 2 mM Cr(III) stress. Zhang and Guo et al. similarly reported increased proline content and antioxidant enzyme activity after NaHS pretreatment in foxtail millet seedlings under salt stress and Salix matsudana Koidz under cadmium stress.^9,39^ AsA and GSH, two antioxidants, directly eliminate ROS through the ASA-GSH cycle, with GR playing a vital role in maintaining AsA and GSH redox balance.^46^ Analysis of GSH and AsA contents and GR enzyme activity revealed the highest GSH level and lowest AsA level at 0.05–0.10 mM NaHS immersion. Additionally, GR enzyme activity at NaHS immersion concentrations of 0.02 mM, 0.05 mM, 0.10 mM, and 0.15 mM was significantly higher than the control (*p* < 0.05), indicating increased non-enzymatic ROS scavenging system activity under 2 mM Cr(III) stress. Thus, we hypothesize that exogenous H_2_S signaling may enhance alfalfa plant survival under 2 mM Cr(III) stress by activating both ROS scavenging enzymatic and non-enzymatic systems to counteract oxidative damage and maintain intracellular redox balance.

H_2_S may improve photosynthetic electron transport efficiency, potentially reducing ROS generation,^47^ and upregulating antioxidant enzyme expression.^48^ This could contribute to improved ROS metabolism balance and redox homeostasis maintenance. Yu et al. studied trivalent Cr(III)-treated rice plants, reporting Cr primarily accumulated in roots;^49^ furthermore, they identified 10 specific OsMT genes regulating ROS removal with differential expression.^50^ CYTc levels decreased in roots due to metal ion binding and increased in shoots, possibly due to ROS accumulation, serving as a signal for severe growth inhibition.^51^ Additionally, cytochrome c oxidase (COX) gene family members, including COX1, COX2, COX5B, COX6A, COX6B, and COX7A, exhibited differential expression.^52^ However, further investigation is needed to understand the molecular mechanism by which H_2_S affects the antioxidant system of alfalfa under Cr(III) stress.

## 5. Conclusion

In conclusion, exposure to Cr(III) stress led to delayed or inhibited seed germination and affected root elongation and root tip development in alfalfa. Presoaking alfalfa seeds with NaHS before planting was observed to alleviate the delayed germination and inhibited root elongation of alfalfa seeds under 2 mM Cr(III) stress. Subsequent investigations indicated that this effect was linked to the activation of intracellular antioxidant enzyme systems and non-enzymatic systems, which function to defend against oxidative damage and maintain the intracellular redox balance in alfalfa seedlings. Considering the role of NaHS, it is suggested that pretreating alfalfa seeds with 0.05 mM NaHS could effectively promote seed germination, enhance root elongation, and maintain the balance of ROS metabolism under 2 mM Cr(III) stress.
